# Complete Genome Sequence of a Hepatitis E Virus Genotype 1e Strain from an Outbreak in Nigeria, 2017

**DOI:** 10.1128/MRA.01378-18

**Published:** 2019-01-03

**Authors:** Olusola Anuoluwapo Akanbi, Dominik Harms, Bo Wang, Oluyinka Oladele Opaleye, Olufisayo Adesina, Folakemi Abiodun Osundare, Abiodun Ogunniyi, Dhamari Naidoo, Isabelle Devaux, Alemu Wondimagegnehu, Clement Peter, Okudo Ifeanyi, Opeayo Ogundiran, Uzoma Ugochukwu, Nwando Mba, Sunday A. Omilabu, Chikwe Ihekweazu, C.-Thomas Bock

**Affiliations:** a Division of Viral Gastroenteritis and Hepatitis Pathogens and Enteroviruses, Department of Infectious Diseases, Robert Koch Institute, Berlin, Germany; b Ladoke Akintola University of Technology, Ogbomoso, Oyo State, Nigeria; c Nigeria Centre for Disease Control, Jabi, Abuja, Nigeria; d Infectious Hazard Management Department, World Health Organization, Geneva, Switzerland; e World Health Organization Nigeria, Abuja, Nigeria; f Department of Medical Microbiology and Parasitology, College of Medicine of the University of Lagos, Lagos, Nigeria; g Institute of Tropical Medicine, University of Tübingen, Tübingen, Germany; Broad Institute of MIT and Harvard University

## Abstract

Hepatitis E virus genotype 1 (HEV-1) is associated with large epidemics. Notably, HEV subtype 1e (HEV-1e) has caused HEV outbreaks in sub-Saharan Africa. We report here the second full-length genome sequence of an HEV-1e strain (NG/17-0503) from a recent outbreak in Nigeria in 2017. It shares 94.2% identity with an HEV-1e strain from Chad.

## Abstract (precis)

Hepatitis E virus genotype 1 (HEV-1) is associated with large epidemics. Notably, HEV subtype 1e (HEV-1e) has caused HEV outbreaks in sub-Saharan Africa.

## ANNOUNCEMENT

Hepatitis E virus (HEV) is the most common causative agent of acute viral hepatitis. Eight HEV genotypes (HEV-1 to HEV-8) have been described, of which five are well recognized as human pathogens (1). HEV-1 and HEV-2 are waterborne, transmitted through fecal-oral routes, and are responsible for large HEV outbreaks in resource-limited countries. HEV-3 and HEV-4 are linked to zoonotic transmission and cause sporadic infections in industrialized countries. It has been reported that HEV subtype 1e (HEV-1e) was responsible for a large outbreak in sub-Saharan Africa (Chad). The T3 strain (GenBank accession no. AY204877) was obtained from an infected person from France during an outbreak in Chad in 1983 and represents the only complete genome sequence of HEV-1e available (2). We report the second full-length genome sequence of an HEV-1e strain (NG/17-0503) from a recent outbreak in Nigeria in 2017. The virus was isolated from a 35-year-old resident of the Mobbar local government area of Borno State, Nigeria. The patient’s serum tested positive for HEV antibodies using Wantai HEV IgM rapid test and Wantai HEV IgM enzyme-linked immunosorbent assay (ELISA; Sanbio, The Netherlands).

Viral RNA was extracted from serum using the QIAamp viral RNA minikit with the QIAcube BioRobot workstation (Qiagen, Hilden, Germany), followed by cDNA synthesis using SuperScript first-strand synthesis (Invitrogen, Carlsbad, CA, USA), according to the manufacturer’s instructions. Real-time reverse transcription-PCR (RT-PCR) targeting the HEV open reading frames (ORF) 2 and 3 showed viremic HEV infection, with a viral load of 2.22 × 10E+6 IU/ml. Sequence analysis of partial ORF1 (307 bp) and ORF2 (401 bp) implemented using the BLASTn search engine (https://blast.ncbi.nlm.nih.gov) indicated that NG/17-0503 had highest nucleotide identity with the T3 strain. Real-time RT-PCR and consensus nested RT-PCR were conducted as previously described (3). The complete viral genome was amplified in fragments using the Kapa HiFi HotStart ReadyMix PCR kit (Roche, Mannheim, Germany) with HEV-1 universal and NG/17-0503 genome-specific primers (Table 1). The 5ʹ and 3ʹ ends were amplified using 5ʹ and 3ʹ rapid amplification of cDNA ends (Roche). All HEV amplicons were sequenced using Sanger sequencing with a BigDye Terminator 3.1 kit (Thermo Fisher, USA) in sense and antisense directions. The whole-genome sequence was assembled and analyzed using Geneious software 10.0.5 (Biomatters Ltd., Auckland, New Zealand) (4). Phylogenetic analyses were performed with the MEGA 7.0.26 software (5).

**TABLE 1:** Primers used for NG/17-0503 complete genome sequencing

Primer^*a*^	Sequence (5′ to 3′)	Location^*b*^
HEV-289_f	CTTGGGCCTTGAGTGTGCTA	4443–4462
HEV-290_f	CCCTATCCAGCGCGTTATACAT	222–243
HEV-291_r	ACCGACAGTAACCTTGTAGCTG	1089–1068
HEV-292_r	CATGAGACGGTCCCAGATATGG	999–978
HEV-293_f	CCTGTGTCGGGTGGAATGAA	5091–5110
HEV-294_r	GGACTGGTCATACTCGGCAG	6595–6576
HEV-295_f	ACGAAGGGTCCGATGTTGAC	1532–1551
HEV-296_r	AATGGCTGGGATCTGGTTCG	3210–3191
HEV-297_r	GACTCTAGCAGCAGTGTGGG	3087–3068
HEV-298_f	GATCCCAGCCATTGACTTCGAA	3198–3219
HEV-299_r	TAGCACACTCAAGGCCCAAG	4462–4443
HEV-300_r	TGTCGTCAAAAGCATCCCCA	4351–4332
HEV-302_f	TGCCACTGTAGAACCATGATCC	1396–1417
HEV-303_r	GGTAGATAAAGCTCATCCCCGG	2771–2750
HEV-304_r	GCGTCAAAACTAGGACCGATTG	2729–2708
HEV-311_f	GTGCTATTATGGAGGAGTGCGG	4457–4478
HEV-312_r	AGCACTATCGAATCATCACCTT	4694–4673
HEV-321_r	ACAGAGCATAACAAGGCCAGAA	5983–5962
HEV-322_f	ATGCTGTTGGTGGCTATGCTAT	5745–5766
HEV-323_r	CCTGGATAACTACACGGGATTCC	6470–6448
HEV-324_f	CATATCCGGGTCCTATGTGGTAC	1575–1597
HEV-325_r	GCATCAACYTCCGACCAAGT	2156–2137
HEV-326_f	GCATGTYTGGGAGTCGGC	2082–2099

a Forward primer designations end with _f; reverse primer designations end with _r.

b Numbering is according the HEV prototype strain Burma (GenBank accession no. M73218).

**Fig 1:**
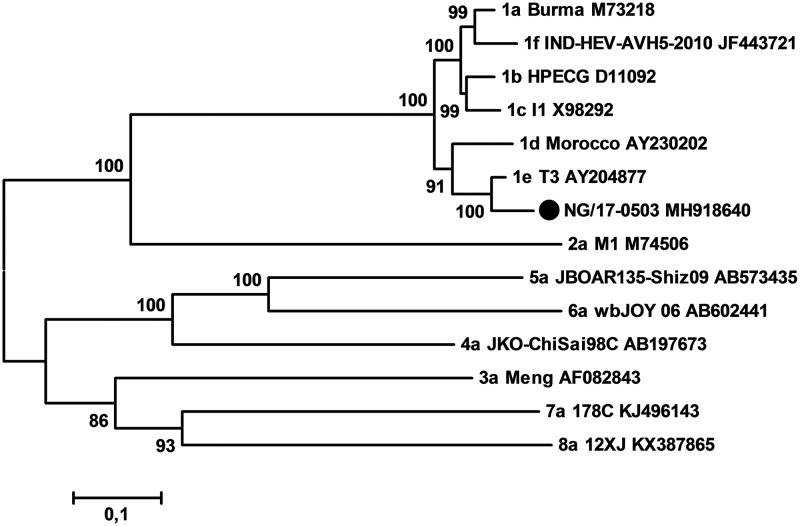
Phylogenetic tree based on complete genome sequences of representative proposed HEV reference strains. Each branch is labeled with the subtype designation, the strain name, and the GenBank accession number. The Nigerian NG/17-0503 strain in this study is marked with a solid circle. A maximum likelihood method based on the general time-reversible model with gamma distributed with invariant sites was inferred. The values at nodes indicate the bootstrap values (using 1,000 replications).

The complete genome of NG/17-0503 is 7,284 nucleotides in length, excluding the poly(A) tail, with a G+C content of 57.6%. The genome contains three ORFs (ORF1, 1,693 amino acids; ORF2, 660 amino acids; ORF3, 123 amino acids) encoding the HEV viral proteins. NG/17-0503 shared the highest identity of 94.2% with the T3 strain from Chad, followed by 88.7% with an HEV-1d Moroccan strain (GenBank accession no. MH918640). Sequences were aligned using the MAFFT algorithm (6). Phylogenetically, NG/17-0503 and T3 formed a separate subcluster within HEV-1 (Fig. 1).

In conclusion, we identified and characterized the full-length genome of an HEV-1e strain circulating during an outbreak in Nigeria in 2017. Sequence and phylogenetic analyses showed that NG/17-0503 is the second full-length HEV-1e genome available to date. Since the Chad HEV-1e strain from 1983 was from a neighboring country of Nigeria, the detection of NG/17-0503 is strongly suggestive of local transmission of this endemic virus over decades.

### Data availability.

The complete genome sequence of NG/17-0503 has been deposited in the GenBank database under the accession no. MH918640.
